# Inunction in Scarlet Fever

**Published:** 1893-09-23

**Authors:** 


### INUNCTION IN SCARLET FEVER.

Mr. J. Brendon Curgenven, M.R.C.S. and L.S.A., writes from Teddington Hall: I had not intended to answer Mr. Kingzett's last letter, but fearing that some of your readers may have misunderstood my argument, as he has done, I must point out that my contention has been as to the suitability or not of "Sanitas" oil as an antiseptic for inunction, and I have not "shifted" my "ground," as Mr. Kingzett asserts. He has stated that "Sanitas" oil contains only "50 per cent. of volatile constituents," the other 50 per cent. would remain on the skin as a varnish. As I have shown you, Sir, it has remained unaltered on paper for two years. I cannot understand how Mr. Kingzett can for one moment think such a substance suitable as a disinfectant for inunction for sprinkling over linen, clothes, carpets, &c., as is necessary in carrying out the method of disinfection of infectious diseases which I advocate. No disinfectant can be suitable for such a purpose unless it is entirely and rapidly volatile, as I assert the "Oleusaban" eucalyptus is.

